# Immunomodulatory Monoclonal Antibodies in Combined Immunotherapy Trials for Cutaneous Melanoma

**DOI:** 10.3389/fimmu.2017.01024

**Published:** 2017-08-25

**Authors:** Mariana Aris, José Mordoh, María Marcela Barrio

**Affiliations:** ^1^Centro de Investigaciones Oncológicas - Fundación Cáncer, Buenos Aires, Argentina; ^2^Instituto Médico Especializado Alexander Fleming, Buenos Aires, Argentina; ^3^Fundación Instituto Leloir, IIBBA-CONICET, Buenos Aires, Argentina

**Keywords:** monoclonal antibodies, immune checkpoint blockade, combined tumor immunotherapy, clinical trials, cutaneous melanoma

## Abstract

In the last few years, there has been a twist in cancer treatment toward immunotherapy thanks to the impressive results seen in advanced patients from several tumor pathologies. Cutaneous melanoma is a highly mutated and immunogenic tumor that has been a test field for the development of immunotherapy. However, there is still a way on the road to achieving complete and long-lasting responses in most patients. It is desirable that immunotherapeutic strategies induce diverse immune reactivity specific to tumor antigens, including the so-called neoantigens, as well as the blockade of immunosuppressive mechanisms. In this review, we will go through the role of promising monoclonal antibodies in cancer immunotherapy with immunomodulatory function, especially blocking of the inhibitory immune checkpoints CTLA-4 and PD-1, in combination with different immunotherapeutic strategies such as vaccines. We will discuss the rational basis for these combinatorial approaches as well as different schemes currently under study for cutaneous melanoma in the clinical trials arena. In this way, the combination of “push and release” immunomodulatory therapies can contribute to achieving a more robust and durable antitumor immune response in patients.

## Introduction

An important role for the immune system in cancer biology has been proposed for decades. However, immunotherapy has been very recently recognized as an effective treatment to control tumor growth and dissemination. Cutaneous melanoma (CM) is a highly mutated and immunogenic tumor and has been a rich field for the development of tumor immunology and immunotherapy (1–3). There is strong evidence of common tumor antigens (Ags) as well as patient’s own tumor neoantigens (neoAgs) which are recognized by the immune system (4); of tumor infiltration by specific immune populations and their clinical correlation (5); and of tumor immunoediting including immune escape strategies (6). More recently, the discovery of multiple immune checkpoint mechanisms, either stimulatory or inhibitory pathways of the immune system, which can be targeted with monoclonal antibodies (mAbs), has burst into intense clinical research. In particular, immune checkpoint blockade (ICKB) with mAbs immunotherapy has proved for the first time to improve overall survival (OS) of CM metastatic patients (7). Nowadays, immunotherapeutic approaches including ICKB with mAbs is the fourth cancer treatment modality along with surgery, radiotherapy, and chemotherapy/targeted therapy. The use of ICKB has expanded beyond CM and these therapies are now approved for the treatment of several metastatic tumors, including renal cell carcinoma, non-small-cell lung carcinoma, squamous cell carcinoma of the head and neck, urothelial carcinoma, and Hodgkin lymphoma (www.fda.gov/drugs).

The inhibitory immune checkpoint molecules act as physiological brakes that prevent potentially harmful immune responses and autoimmunity. Cytotoxic T-lymphocyte antigen 4 (CTLA-4) is constitutively expressed in regulatory T cells (Tregs) but is only upregulated in T-cells after activation. Physiologically, CTLA-4 transmits an inhibitory signal to T cells to shut down immune responses by interaction with CD80 and CD86 molecules, which are expressed at the surface of Ag-presenting cells (APCs) such as dendritic cells (DCs) and macrophages; CTLA-4 also contributes to the inhibitory function of Tregs (8). Ipilimumab (Bristol–Myers Squibb) targets CTLA-4, blocking the inhibitory signal, unleashing cytotoxic T cells to eliminate the cancer cells (7). Another inhibitory immune-checkpoint that has been extensively targeted with mAbs is the axis programmed cell death-1/CD279 (PD-1) and their ligands PD-L1 (B7-H1/CD274) and PD-L2 (B7-DC/CD273) (9). PD-1 is expressed on the membrane of activated T lymphocytes; PD-L1 and PD-L2 are expressed in APCs and also in some tumor cells. PD-1 and PD-L1/PD-L2 binding induce a coinhibitory signal that limits the development of the T-cell response. Several blocking mAbs targeting PD-1 are currently indicated to treat different tumors, such as nivolumab (Bristol–Myers Squibb) (10) and pembrolizumab (Merck Sharp & Dohme) (11). Accordingly, there are mAbs targeting PD-L1, such as atezolizumab (Roche), avelumab (EMD, Serono), and durvalumab (Astra Zeneca). Blocking of CTLA-4 molecule would affect the initial priming phase while targeting the PD-1/PD-L1 axis would interfere more profoundly with the effector phase of the anti-immune response. Although ICKB has shown to potentiate long-lasting antitumor immune response in the metastatic setting, about only 30% of patients achieve durable responses to ICKB with mAbs, thus intense research is ongoing to unravel the mechanisms involved in both primary and acquired ICKB resistance.

**Figure 1:**
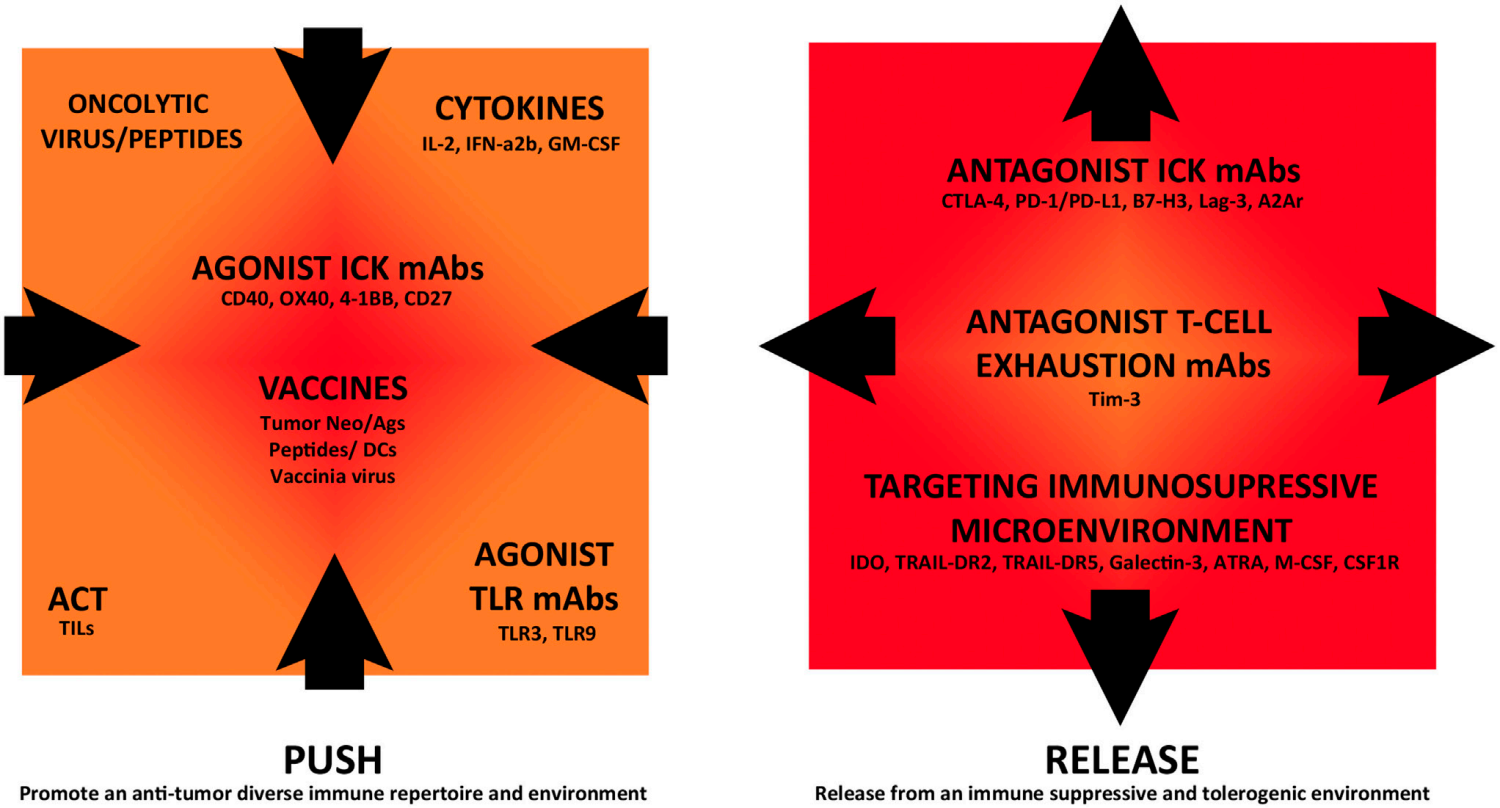
Immunomodulatory monoclonal antibodies and combination strategies in cutaneous melanoma immunotherapy. Different immunotherapeutic strategies are currently being assessed in combination in clinical trials that either “push” tumor immunoreactivity or “release” from inhibitory immune-regulatory mechanisms, fostering this way an antitumor immune response.

Now that ICKB has proven to induce clinical responses for several tumor pathologies, it is currently being investigated in combination with other immunomodulatory strategies in clinical trials, in an attempt to improve antitumor immune responses. ICKB can be combined with antagonists of immunosuppressive molecules or different immunostimulatory treatments, such as active tumor vaccines, administration of cytokines, tumor-specific mAbs, and adoptive cell therapy (ACT). As shown in Figure 1, future immunotherapy can be seen from an integrative point of view, allowing the combination of different approaches that target both the tumor cells and the immune microenvironment, with impact in the systemic immune status. In this review, we have selected several examples of such combinatory strategies that are currently being investigated in clinical studies to discuss the rationale and potential results (www.clinicaltrials.gov) (Tables 1–3). Most of these trials are in the initial phases (I–II) and are directed toward advanced metastatic tumor patients including CM, mainly to optimize dose regimens and observe safety, side effects, and initial response in patients, including potential immunogenicity and antitumor immune response.

**Table 1:** Clinical trials combining immune checkpoint blockade with immunostimulatory strategies.

Trial identifier/study phase/status	Combination therapy	Patient condition	Sponsor	Official study title	Study design
NCT02515227 Phase I/II (recruiting, 2015)	• Pembrolizumab (antagonist PD-1, mAb) • 6MHP peptide vaccine (6 class II MHC-restricted helper peptides)	Metastatic melanoma (MM)	Craig L Slingluff, Jr	A trial to evaluate the safety, immunogenicity, and clinical activity of a helper peptide vaccine plus PD-1 blockade	6MHP vaccine (200 mg/each six peptides), mixed 1/1 with Montanide ISA-51, will be administered intradermally (ID)/subcutaneous (SC) at days 1, 8, 15, 43, 64, and 85. Pembrolizumab 200 mg intravenous (IV)/3 weeks/2 years

NCT02385669 Phase I/II (recruiting, 2015)	• Ipilimumab [antagonist (CTLA-4) mAb] • 6MHP peptide vaccine (6 class II MHC-restricted helper peptides)	MM	Craig L Slingluff, Jr	A Phase I/II trial to evaluate the safety, immunogenicity, and clinical activity of a helper peptide vaccine plus CTLA-4 blockade in advanced melanoma (Mel62; 6PAC)	6MHP vaccine (200 mg/each six peptides), mixed 1/1 with Montanide ISA-51, will be administered ID/SC at days 1, 8, 15, 43, 64, and 85. Ipilimumab will be administered 3 mg/kg IV/3 weeks/four doses

NCT03047928 Phase I/II (recruiting, 2017)	• Nivolumab (antagonist PD-1 mAb) • PD-L1/indoleamine 2,3-dioxygenase (IDO) peptide vaccine	MM	Inge Marie Svane	Combination therapy with nivolumab and PD-L1/IDO peptide vaccine to patients with MM	• Patients receive nivolumab IV 3 mg/kg biweekly until progression. • Vaccine administration starts concomitantly with nivolumab, biweekly six times, then every fourth week up 1 year. A vaccine consists of 100 µg IDO peptide, 100 µg PD-L1 peptide, and 500 μl Montanide as adjuvant. • Patients who complete all vaccines will continue nivolumab treatment after standard guidelines

NCT01176461 Phase I (ongoing, not recruiting, 2010)	• BMS-936558: antagonist PD-1 mAb • Peptide vaccine: MART-1, NY-ESO-1, gp100 • Adjuvant: Montanide ISA 51 VG	MM	H. Lee Moffitt Cancer Center, National Cancer Institute (NCI), Bristol–Myers Squibb, Medarex	A pilot trial of a vaccine combining multiple class I peptides and Montanide ISA 51 VG with escalating doses of anti-PD-1 antibody BMS-936558 for patients with unresectable Stages III/IV melanoma	• Arm 1: Phase I dose escalation cohort. Six doses of BMS-936558 and six peptide vaccines administered/2 weeks/12 weeks. • Arm comparator: BMS-936558 without peptide vaccine

NCT02897765 Phase I (recruiting, 2016)	• NEO-PV-01 personalized vaccine • Nivolumab • Poly-ICLC	Metastatic tumors including MM	Neon Therapeutic Inc., Bristol–Myers Squibb	An open-label, Phase IB study of NEO-PV-01 + adjuvant with nivolumab in patients with melanoma, non-small-cell lung carcinoma or transitional cell carcinoma of the bladder	• Nivolumab 240 mg IV infusion/2 weeks. Patients who have not achieved a CR to nivolumab alone at week 12 will receive NEO-PV-01 + adjuvant SC (one vial of pooled peptides per injection site) in up to four distinct sites (each extremity or flanks) while continuing therapy with nivolumab

NCT01302496 Phase II (completed, 2017)	• TriMix-DC vaccine • ipilimumab (antagonist CTLA-4 mAb)	MM	Bart Neyns, Vrije Universiteit Brussel	A two-stage Phase II study of autologous TriMix-DC therapeutic vaccine in combination with ipilimumab in patients with previously treated unresectable stage III or IV melanoma	Patients will receive five TriMix-DC doses. All administrations but first will be preceded by ipilimumab 10 mg/kg. NED patients will be offered ipilimumab maintenance (10 mg/kg q12wks)

NCT02432963 Phase I (recruiting, 2015)	• Pembrolizumab (antagonist PD-1 mAb) • modified vaccinia virus ankara vaccine expressing p53	Metastatic tumors including MM	City of Hope Medical Center	A Phase I study of a p53MVA vaccine in combination with pembrolizumab	Patients receive pembrolizumab IV followed by p53MVA Vaccine at least 30 min later once in weeks 1, 4, 7

NCT02275416 Phase I/II (recruiting, 2014)	• Ipilimumab (antagonist CTLA-4 mAbs). • Biological: UV1 vaccine (peptide based-vaccine directed to hTERT) • Biological: GM-CSF	MM	Ultimovacs AS	Safety of UV1 vaccination in combination with ipilimumab in patients with unresectable or metastatic malignant melanoma	• Ipilimumab (3 mg/kg)/3 weeks/four doses. UV1 vaccine 300 µg plus GM-CSF 75 µg ID in the lower abdomen every 4 weeks up to 28 weeks, at weeks 36 and 48

NCT00058279 Phase I/II (completed, 2006)	• Ipilimumab (antagonist CTLA-4 mAb) • Aldesleukin (IL-2)	Intraocular/skin MM	NCI	MDX-CTLA4 combined with IL-2 for patients with MM	Patients received 3.0 mg/kg ipilimumab every 3 weeks and IL-2 (720,000 IU/kg every 8 h to a maximum of 15 doses)

NCT02983045 Phase I/II (recruiting, 2016)	• Nivolumab (antagonist PD-1 mAb) • NKTR-214 (IL-2)	Metastatic tumors including MM	Nektar Therapeutics	A Phase 1/2, open-label, multicenter, dose escalation and dose expansion study of NKTR-214 and nivolumab in patients with select locally advanced or metastatic solid tumor malignancies	• Phase 1: patients will receive NKTR-214 every 14/21 days, in combination with 240 mg/360 mg nivolumab every 14/21 days. • Phase 2: additional patient cohorts will be dosed at the recommended Phase 2 dose/schedule of NKTR-214 and nivolumab (as determined by Phase 1 of the trial)

NCT02748564 Phase I/II (recruiting, 2017)	• Pembrolizumab (antagonist PD-1 mAb) • aldeleuskin (IL-2)	Metastatic tumors including MM	Rutgers, The State University of New Jersey	A Phase 1b/II trial of interleukin-2 in combination with pembrolizumab for patients with unresectable or MM	Patients will receive pembrolizumab IV every 3 weeks and aldesleukin IV every 8 h for up to 14 doses at weeks 4, 7, 16, 19, 28, and 31 in the absence of progression/toxicity

NCT01608594 Phase I (ongoing, not-recruiting, 2013)	• Ipilimumab (antagonist CTLA-4 mAb) • HDI (high-dose IFN-a2b)	MM	Ahmad Tarhini	Neoadjuvant combination biotherapy with ipilimumab (3 or 10 mg/kg) and high-dose IFN-α2B in patients with locally/regionally advanced/recurrent melanoma: a randomized safety, efficacy and biomarker study	Patients will receive IFN-a2b at 20 MU/m^2^/day IV for 5 consecutive days/4 weeks, followed by 10 MU/m^2^/day SC thrice a week/2 weeks, followed by definitive surgery. After recovery, IFN-a2b will be resumed at 10 MU/m^2^/day SC, thrice a week/46 additional weeks. IFN-a2b will be given concurrently with ipilimumab at 3 or 10 mg/kg

NCT01708941 Phase II (ongoing, not-recruiting, 2013)	• Ipilimumab (antagonist CTLA-4 mAb) • HDI (high-dose IFN-a2b)	MM	NCI	A randomized Phase II study of ipilimumab at 3 or 10 mg/kg alone or in combination with high-dose interferon-alpha in advanced melanoma	There are different cohorts where patients either receive higher dose ipilimumab, higher dose ipilimumab plus HDI, lower dose ipilimumab, and lower dose ipilimumab plus HDI

NCT02112032 Phase I (recruiting, 2014)	• MK-3475 (antagonist PD-1 mAb) • PegIFN-a2b	MM	Hassane M. Zarour, MD	Phase 1 study of anti-PD-1 antibody MK-3475 and PegIFNa-2b for advanced melanoma	2 years treatment with MK-3475: 2 mg/kg every 3 weeks IV and PegIFN-a2b: 1 µg/kg every week SC

NCT02339324 Phase I (recruiting, 2015)	• Pembrolizumab (antagonist PD-1 mAb) • HDI	MM	University of Pittsburgh	Neoadjuvant combination biotherapy with pembrolizumab and high dose IFN-alfa2b in patients with locally/regionally advanced/recurrent melanoma: safety, efficacy and biomarker study	• Induction phase (first 6 weeks): pembrolizumab IV for two doses/4 weeks concurrently with HDI IV × 5 consecutive days/week/4 weeks, followed by SC every other day 3× each week/2 weeks. • Surgery phase (week 6–8). • Maintenance phase (following recovery from surgery): pembrolizumab IV infusion/3 weeks given concurrently with HDI SC every week/46 additional weeks

NCT02174172 Phase I (recruiting, 2014)	• Atezolizumab (antagonist PD-L1 mAb) • bevacizumab (antagonist VEGF mAb)• ipilimumab (antagonist CTLA-4 mAb) • obinutuzumab (antagonist CD20 mAb) • IFNa2b • Peg-IFNa2b	Metastatic tumors including MM	Hoffmann-La Roche	A Phase Ib study of the safety and pharmacology of atezolizumab (anti-PD-L1 antibody) administered with ipilimumab, interferon-alpha, or other immune-modulating therapies in patients with locally advanced or metastatic solid tumors	• Arm A: atezolizumab with ipilimumab; • Arm B: atezolizumab with IFN-A2b; • Arm C: Atezolizumab With PEG-IFN-A2b; • Arm D: atezolizumab with PEG-IFN-A2b and bevacizumab; • Arm E: atezolizumab with obinutuzumab

NCT02009397 Phase I/II (recruiting, 2012)	• Ipilimumab (antagonist CTLA-4 mAb) • rhuGM-CSF	MM	J Graham Brown Cancer Center	A Phase I/II open-label study of ipilimumab and GM-CSF administered to unresectable Stage IIIC and Stage IV melanoma patients	IV ipilimumab followed by SC GM-CSF, for up to four cycles

NCT02652455 Phase I (recruiting, 2016)	• Nivolumab (PD-1 antagonist mAb) • autologous tumor infiltrating lymphocyte (TIL) • CD137 agonist mAb • cyclophosphamide • fludarabine • IL-2	MM	H. Lee Moffitt Cancer Center and Research Institute	A pilot clinical trial combining PD-1 blockade, CD137 agonism and adoptive cell therapy for MM	• Patients will receive treatment with nivolumab prior to tumor removal for TIL growth. • Surgery and TIL growth *ex vivo*. • Patients lymphodepleting chemotherapy with cyclophosphamide and fludarabine; TIL infusion; interleukin-2 treatment. • The first six participants will not receive nivolumab prior treatment for comparison

NCT01701674 Phase I (ongoing, not-recruiting, 2012)	• Ipilimumab (CTLA-4 antagonist mAb) • autologous TIL • cyclophosphamide • IL-2	MM	H. Lee Moffitt Cancer Center and Research Institute	Costimulation with ipilimumab to enhance lymphodepletion plus adoptive cell transfer and high dose IL-2 in patients with MM	Combination of ipilimumab followed by lymphodepletion with chemotherapy, TIL infusion, and high dose IL-2

NCT02027935 Phase II (recruiting, 2015)	• Ipilimumab (CTLA-4 antagonist mAb) • autologous CD8 • cyclophosphamide • IL-2	MM	M.D. Anderson Cancer Center	Phase II study of cellular adoptive immunotherapy using autologous CD8^+^ antigen-specific T cells and anti-CTLA4 for patients with MM	• Patients’ leukapheresis to get and cultivate CD8^+^ T cells. • Cyclophosphamide 300 mg/m^2^ for lymphodepletion. • IV Infusion of 10^10^ T cells/m^2^. • IL-2 250,000 U/m^2^ SC every 12 h for 14 days. • Ipilimumab 3 mg/kg IV 24 h postinfusion and days 22, 43, and 64

NCT03123783 Phase I/II (recruiting, 2017)	• Ipilimumab (antagonist CTLA-4 mAb) • APX005M (agonist CD40 mAb)	MM	Apexigen, Inc.	A study to evaluate the safety and efficacy of the CD40 agonistic antibody APX005M administered in combination with nivolumab in subjects with non-small-cell lung cancer and subjects with MM	Subjects will receive intravenously APX005M in combination with nivolumab until disease progression, unacceptable toxicity or death

NCT02706353 Phase I/II (recruiting, 2017)	• Pembrolizumab (antagonist PD-1 mAb) • APX005M (agonist CD40 mAb)	Metastatic tumors including MM	M.D. Anderson Cancer Center	Phase I/II dose escalation and cohort expansion of safety and tolerability study of intratumoral CD40 agonistic monoclonal antibody APX005M in combination with systemic pembrolizumab in patients with MM	• Dose escalation phase: initial dose APX005M 0.1 mg injected into 1–3 tumors every 3 weeks/four doses up to maximum tolerated dose (MTD). All participants will receive pembrolizumab IV 2 mg/kg/3 weeks. • Dose expansion phase: APX005M MTD, same of pembrolizumab dosage

NCT02554812 Phase II (ongoing, 2015)	• Avelumab (antagonist PD-L1 mAb) • utomilumab (agonist 41BB mAb) • PF-04518600 (agonist OX-40 mAb) • PD 0360324 (neutralizing M-CSF mAb)	Metastatic tumors including MM	Pfizer	A Phase 1b/2 open-label study to evaluate safety, clinical activity, pharmacokinetics and pharmacodynamics of avelumab (msb0010718c) in combination with other cancer immunotherapies in patients with advanced malignancies	• Arm A: avelumab + utomilumab at three different dose levels. • Arm B: dose escalation PF-04518600 + avelumab. • Arm C: dose escalation PD 0360324 + avelumab. • Arm D: dose escalation utomilumab + PF-04518600 + avelumab. Afterward, dose expansion utomilumab + PF-04518600 + avelumab in selected tumor types

NCT02643303 Phase I/II (recruiting, 2016)	• Durvalumab (antagonist PD-1 mAb) • Tremelimumab (antagonist CTLA-4 mAb) • poly ICLC (TLR3 agonist molecule)	Metastatic tumors including MM	Ludwig Institute for Cancer Research	A Phase 1/2 study of *in situ* vaccination with tremelimumab and IV durvalumab (MEDI4736) plus the toll-like receptor (TLR) agonist PolyICLC in subjects with advanced, measurable, biopsy-accessible cancers	• Phase I, cohort A: IV durvalumab + IT/IM polyICLC; cohort B: tremelimumab + IT/IM polyICLC; cohort C: durvalumab + tremelimumab + IT/IM polyICLC. Phase II: once the recommended combination has been determined, subsequent subjects will follow this dosing scheme

NCT02644967 Phase I/II (recruiting, 2015)	• Ipilimumab (antagonist CTLA-4 mAb) • pembrolizumab (antagonist PD-1 mAb) • IMO-2125 (TLR-9 agonist)	MM	Idera Pharmaceuticals, Inc.	A Phase 1/2 study to assess the safety and efficacy of intratumoral IMO-2125 in combination with ipilimumab or pembrolizumab in patients with MM	• Cohort 1: IMO-2125 IT weekly, then once/3 weeks. Ipilimumab IV at 3 mg/kg • Cohort 2: IMO-2125, IMO-2125 IT weekly, then once/3 weeks. Pembrolizumab, IV at 2 mg/kg/3 weeks

NCT02668770 Phase I (recruiting, 2016)	• Ipilimumab (antagonist CTLA-4 mab) • MGN1703 (TLR-9 Agonist molecule)	Metastatic tumors including MM	M.D. Anderson Cancer Center	A Phase I trial of ipilimumab (immunotherapy) and MGN1703 (TLR agonist) in patients with advanced solid malignancies	Dose escalation and expansion group of MGN1703 doses, SC or ID ipilimumab will be administrated 3 mg/kg/cycle 8 days following MGN1703 administration

NCT02981303 Phase II (recruiting, 2016)	• Pembrolizumab (antagonist PD-1 mAb) • Imprime PGG (PAMP)	Metastatic tumors including MM	Biothera	A multicenter, open-label, Phase 2 study of imprime PGG and pembrolizumab in subjects with advanced melanoma failing front-line treatment with checkpoint inhibitors or triple negative breast cancer failing front-line chemotherapy for metastatic disease	• Imprime PGG IV 4 mg/kg on days 1, 8, 15/3-week treatment cycle. • Pembrolizumab IV 200 mg/kg following Imprime infusion

**Table 2:** Clinical trials combining immune checkpoint blockade with targeting of immunosuppressive molecules.

Trial identifier/study phase/status	Combination therapy	Patient condition	Sponsor	Official study title	Study design
NCT02743819 Phase II (recruiting, 2016)	• Pembrolizumab (antagonist PD-1 mAb) • ipilimumab [antagonist (CTLA-4) mAb]	Metastatic melanoma (MM)	University of Chicago	Phase II study of pembrolizumab and ipilimumab following initial anti-PD1/L1 antibody	Pembrolizumab plus ipilimumab. • Arm A: progression on anti-PD1/L1 antibody • Arm B: stable disease more than 24 weeks or initial response on anti-PD1/L1 antibody

NCT02381314 Phase I (recruiting, 2015)	• Ipilimumab (antagonist CTLA-4 mab) • enoblituzumab (B7-H3 mAb)	Metastatic tumors including MM	MacroGenics	A Phase 1, open-label, dose escalation study of MGA271 in combination with ipilimumab in patients with melanoma, non-small-cell lung cancer, and other cancers	Enoblituzumab will be administered IV once/week (51 doses) to determine maximum tolerated dose (MTD) in combination with ipilimumab, which is administered IV/3 weeks/four doses

NCT02460224 Phase I/II (recruiting, 2015)	• PDR001 (antagonist PD-1 mAb) • LAG525 (antagonist LAG-3 mAb)	Metastatic tumors including MM	Novartis Pharmaceuticals	A Phase I/II, open label, multicenter study of the safety and efficacy of LAG525 single agent and in combination with PDR001 administered to patients with advanced malignancies	• Arm A: LAG525 single treatment arm. • Arm B: LAG525 plus PDR001 combination arm. • Arm C: LAG525 single treatment arm in Japanese patients

NCT02655822 Phase I (recruiting, 2016)	• CPI-444 (blocking adenosine-A2A receptor inhibitor) • atezolumab (antagonist PD-L1 mAb)	Metastatic tumors including MM	Corvus Pharmaceuticals, Inc.	A Phase 1/1b, open-label, multicenter, repeat-dose, dose-selection study of CPI-444 as single agent and in combination with atezolizumab in patients with selected incurable cancers	• Cohort I: CPI-444 100 mg orally twice daily for the first 14 days/each 28-day cycle. • Cohort II: CPI-444 100 mg orally twice daily for 28 days/each 28-day cycle. • Cohort III: CPI-444200 mg orally once daily for the first 14 days/each 28-day cycle. • Cohort IV: CPI-444 MTD + atezolizumab IV

NCT02817633 Phase I (recruiting, 2016)	• Antagonist PD-L1 mAb • TSR-022 (antagonist Tim-3 mAb)	Metastatic tumors including MM	Tesaro, Inc.	A Phase 1 dose escalation and cohort expansion study of TSR-022, an Anti-TIM-3 monoclonal antibody, in patients with advanced solid tumors	• Part 1: Dose Escalation. 1a: dose escalation TSR-022 alone. 1b: dose escalation TSR-022 plus anti-PD-1 antibody. 1c: Phase 2 TSR-022 MTD plus anti-PD-1 antibody. • Part 2: expansion cohorts of Part 1

NCT02608268 Phase I/II (recruiting, 2015)	• PDR001 (antagonist PD-1 mAb) • MBG453 (antagonist Tim-3 mAb)	Metastatic tumors including MM	Novartis Pharmaceuticals	Phase I–Ib/II open-label multi-center study of the safety and efficacy of MBG453 as single agent and in combination with PDR001 in adult patients with advanced malignancies	• Cohort 1: MBG453 dose escalation. • Cohort 2: MBG453 dose escalation in combination with PDR001

NCT02983006 Phase I (recruiting, 2016)	• Nivolumab (antagonist PD-L1 mAb) • DS-8273a (TRAIL-DR5 mAb)	MM	New York University School of Medicine	A Phase 1 study of TRAIL-DR5 antibody DS-8273a administered in combination with nivolumab in subjects with unresectable Stage III or Stage IV melanoma	DS-8273a: starting dose 4 mg/kg IVQ 3 weeks. Dose Escalation: 8 mg/kg IV Q 3 weeks, 16 mg/kg IV Q 3 weeks, 24 mg/kg IV Q 3 weeks, 2 mg/kg IV Q 3 weeks, 4 mg/kg IV Q 3 weeks. • nivolumab: 5 mg/kg IV Q 3 weeks

NCT02471846 Phase I (recruiting, 2015)	• Atezolizumab (antagonist PD-1 mAb) • GDC-0919 (IDO inhibitor)	Metastatic tumors including MM	Genentech, Inc.	A Phase Ib, open-label, dose-escalation study of the safety and pharmacology of GDC-0919 administered with atezolizumab in patients with locally advanced or metastatic solid tumors	• Relapsed cohorts to PD1/PD-L1 blockade will receive GDC-0919 at MTD. • Untreated advanced patients will receive escalation doses of atezolizumab and GDC-0919 combinations. • An expansion cohort of atezolizumab and GDC-0919 combination at MTD

NCT02318277 Phase I/II (recruiting, 2014)	• Durvalumab (blocking PD-L1 mAb) • epacadostat (IDO-1 inhibitor molecule)	Metastatic tumors including MM (B7H3^+^)	Incyte Corporation	A Phase 1/2 study exploring the safety, tolerability, and efficacy of epacadostat (INCB024360) in combination with durvalumab (MEDI4736) in subjects with selected advanced solid tumors (ECHO-203)	Durvalumab IV at selected dose levels every 2 weeks plus epacadostat 25 mg BID as starting dose, followed by dose escalations until MTD

NCT02327078 Phase I/II (recruiting, 2014)	• Nivolumab (PD-1 antagonist mab) • epacadostat (IDO-1 inhibitor)	Metastatic tumors including MM	Incyte Corporation	A Phase 1/2 study of the safety, tolerability, and efficacy of epacadostat administered in combination with nivolumab in select advanced cancers (ECHO-204)	• Phase 1: nivolumab IV 3 mg/kg/2 weeks plus epacadostat 25 mg BID as starting dose, followed by dose escalations. • Phase 2: nivolumab 240 mg 2 weeks plus epacadostat MTD

NCT02073123 Phase I/II (recruiting, 2014)	• Ipilimumab (antagonist CTLA-4 mAb) • pembrolizumab (antagonist PD-1 mAb) • nivolumab (antagonist PD-1 mAb) • indoximod (IDO inhibitor)	MM	NewLink Genetics Corporation	A Phase 1/2 study of the concomitant administration of indoximod plus immune checkpoint inhibitors (CPIs) for adult patients with advanced or MM	• Indoximod 1,200 mg BID concurrently with ipilimumab IV 3 mg/kg/3 weeks/four doses. • Indoximod 1,200 mg BID and pembrolizumab IV at 2 mg/kg/3 weeks. • Indoximod 1,200 mg BID and nivolumab IV at 3 mg/kg/4 weeks

NCT02117362 Phase I (recruiting, 2014)	• Ipilimumab (antagonist CTLA-4 mAb) • GR-MD-02 (Galectin-3 Inhibitor)	MM	Providence Health & Services	Phase IB study of a galectin inhibitor (GR-MD-02) and ipilimumab in patients with MM	Cohorts with escalating doses of GR-MD-02 (1, 2, 4, 8 mg/kg) 1 hour before 3 mg/kg of ipilimumab on days 1, 22, 43, and 65

NCT02403778 Phase II (ongoing, not-recruiting, 2015)	• Ipilimumab (CTLA-4 antagonist mab) • All-trans retinoic acid (ATRA)	MM	University of Colorado, Denver	Ipilimumab and ATRA combination treatment of Stage IV melanoma	• Arm A: ipilimumab 10 mg/kg/3 weeks/four doses. • Arm B: ipilimumab 10 mg/kg/3 weeks/four doses plus 150 mg/m^2^ ATRA orally for 3 days surrounding ipilimumab dosage

NCT02807844 Phase I/II (recruiting, 2016)	• PDR001 (antagonist PD-1 mAb) • MCS110 (blocking MCSF mAb)	Metastatic tumors including MM	Novartis Pharmaceuticals	A Phase Ib/II, open label, multicenter study of MCS110 in combination with PDR001 in patients with advanced malignancies	MCS110 combined with PDR001

NCT02452424 Phase I/II (recruiting, 2015)	• Pembrolizumab (PD-1 mAb) • PLX3397 (CSF1R inhibitor)	Metastatic tumors including MM	Plexxikon	Phase 1/2a study of double-immune suppression blockade by combining a CSF1R inhibitor (PLX3397) with an Anti-PD-1 antibody (pembrolizumab) to treat advanced melanoma and other solid tumors	• Part 1: open-label, sequential PLX3397 dose escalation with a fixed dose of pembrolizumab (200 mg, IV) in approximately 24 patients with advanced solid tumors. • Part 2: extension cohort

NCT02880371 Phase I/II (recruiting, 2016)	• Pembrolizumab (antagonist PD-1 mAb) • ARRY-382 (CSF1R)	Metastatic tumors including MM	Array BioPharma	A Study of ARRY-382 in combination with pembrolizumab, a programmed cell death receptor 1 (PD-1) antibody, for the treatment of patients with advanced solid tumors	• Part A: escalating doses of ARRY-382 with pembrolizumab 2 mg/kg. • Part B: ARRY-382 at MTD with pembrolizumab 2 mg/kg. • Part C: ARRY-382 at MTD with 200 mg pembrolizumab

**Table 3:** Other combinations in clinical trials with immunomodulatory monoclonal antibodies.

Trial identifier/study phase/status	Combination therapy	Patient condition	Sponsor	Official study title	Study design
NCT01740297 Phase I/II (completed, 2015)	• Ipilimumab (antagonist CTLA-4 mAb) • talimogene laherparepvec (oncolytic virus)	Metastatic melanoma (MM)	Amgen	Phase 1b/2, multicenter, open-label trial to evaluate the safety and efficacy of talimogene laherparepvec and ipilimumab compared to ipilimumab alone in subjects with unresected, stage IIIB-IV melanoma	• Experimental: Phase 1b and Phase 2 Arm 1. Talimogene laherparepvec plus ipilimumab. • Active Comparator: Phase 2 Arm 2. Ipilimumab

NCT02263508 Phase Ib/III (recruiting, 2014)	• Pembrolizumab (antagonist PD-1 mAb) • talimogene laherparepvec (oncolytic virus)	MM	Amgen	A Phase 1b/3, multicenter, trial of talimogene laherparepvec in combination with pembrolizumab (MK-3475) for treatment of unresectable stage IIIB to IVM1c melanoma (MASTERKEY-265/KEYNOTE-034)	• Experimental: Phase 3 Arm 1, talimogene laherparepvec and pembrolizumab (MK-3475). • Experimental: Phase 3 Arm 2: placebo and pembrolizumab (MK-3475)

NCT02272855 phase II (ongoing, not-recruiting, 2014)	• Ipilimumab (agonist CTLA-4 mAb) • HF10 (vaccinia virus)	MM	Takara Bio Inc	A Phase II study of combination treatment with HF10, a Replication-competent HSV-1 oncolytic virus, and ipilimumab in patients with Stage IIIB, Stage IIIC, or Stage IV unresectable or metastatic malignant melanoma	Patients will receive 1.10^7^ TCID50/mL HF10 (four injections/once a week; two injections/once at 3 weeks) and ipilimumab 3 mg/kg IV/3 weeks/four total doses

NCT03003676 Phase I (recruiting, 2016)	• Pembrolizumab (antagonist PD-1 mAb) • ONCOS-102 (oncolytic virus)	MM	Targovax Oy	A pilot study of sequential ONCOS-102, an engineered oncolytic adenovirus expressing GMCSF, and pembrolizumab in patients with advanced or unresectable melanoma progressing after PD1 blockade	Patients will receive three doses of intratumoral (i.t.) injection of ONCOS-102 (days 1, 4, and 8) at 3 × 1011 viral particles (VP), preceded by intravenous (i.v.) cyclophosphamide priming 1–3 days prior to day 1. They will then receive pembrolizumab i.v., 2 mg/kg, on day 22 (week 3) and every 3 weeks thereafter until the end of treatment visit on day 169 (week 24)

NCT01986426 Phase I (recruiting, 2013)	• Ipilimumab (antagonist CTLA-4 mAb) • pembrolizumab (antagonist PD-1 mAb) • LTX-315 (lytic peptide)	Metastatic tumors including MM	Lytix Biopharma AS	A Phase I, open-label, multiarm, multicenter, multi-dose, dose escalation study of LTX-315 as monotherapy or in combination with either ipilimumab or pembrolizumab in patients with transdermally accessible tumors	• Arm A: LTX-315 monotherapy at single/sequential lesions. • Arm B: LTX-315 monotherapy at concurrent multiple lesions. • Arm C: LTX-315 plus ipilimumab in MM patients. • Arm D: LTX-315 plus pembrolizumab in triple-negative breast cancer patients

NCT02302339 Phase II (recruiting, 2016)	• Glembatumumab vedotin (gpNMB conjugate-drug mAb) • varlilumab (CD27 agonist mab) • nivolumab/pembrolizumab (antagonist PD-1 mAb)	MM	Celldex Therapeutics	A Phase 2 study of glembatumumab vedotin, an anti-gpNMB antibody–drug conjugate, as monotherapy or in combination with immunotherapies in patients with advanced melanoma	• Cohort A: glembatumumab vedotin IV on day 1/21 day cycle. • Cohort B: glembatumumab vedotin IV on day 1/21 day cycle. Varlilumab IV on day 1 of cycles 1, 2, 4, 6, 8 and 10. • Cohort C: glembatumumab vedotin IV on day 1/21 day cycle. Nivolumab/pembrolizumab administered according to institutional standard of care

NCT02076633 Phase II (completed, 2015)	• L19IL2 (HDAC4 mab conjugated with IL-2) • L19TNF (HDAC4 mab conjugated with TNF)	MM	Philogen S.p.A.	A Phase II study of intratumoral application of L19IL2/L19TNF in melanoma patients in clinical Stage III or Stage IV M1a with presence of injectable cutaneous and/or SC lesions	Patients will be treated with intratumoral injections of 10 Mio IU L19IL2 and 312 µg L19TNF once weekly for up to 4 weeks

NCT02315066 Phase I (recruiting, 2015)	• PF-04518600 (agonist OX40 mAb) • PF-05082566 (agonist 41BB mAb)	Metastatic tumors including MM	Pfizer	A Phase 1, open-label, dose escalation study of Pf-04518600 as a single agent and in combination with Pf-05082566 in patients with selected locally advanced or metastatic carcinomas	• Part A1—PF-04518600 will be administered IV every 14 days starting at a dose of 0.01 mg/kg, increasing until maximum tolerated dose (MTD) is determined. • Part B1 -PF-04518600 will be administered IV every 2 weeks starting at a dose of 0.1 mg/kg and PF-05082566 will be administered IV 4 weeks starting at a dose of 20 mg. Increases in dose will continue until MTD is determined

NCT02714374 Phase I (recruiting, 2016)	• Eculizumab (C5 neutralizing mab) • GL-ONC1 (vaccinia virus)	Metastatic tumors including MM	Kaitlyn Kelly, MD	An open label, non-randomized Phase 1b study to investigate the safety and effect of the oncolytic virus GL-ONC1 administered intravenously with or without eculizumab prior to surgery to patients with solid organ cancers undergoing surgery for curative-intent or palliative resection	• Arm A: GL-ONC1 escalation dose. • Arm B: GL-ONC1 escalation dose plus eculizumab, single dose on week 1/day 1 at 900 mg 60–90 min prior to GL-ONC1

## Clinical Trials

### ICKB Combined with Immunostimulatory Strategies

#### Vaccines

Therapeutic vaccines are under investigation but still have shown only limited clinical benefit for patients with CM and other tumors. The rationale of therapeutic vaccination is to boost the patient’s immune system to induce a pro-inflammatory T_H_1 immune response targeting both shared common tumor Ags and patient-specific neoAgs generated by somatic mutations in a personalized fashion. Vaccines can be prepared as peptides, tumor lysates, or irradiated whole tumor cells, administered with adjuvants to potentiate the immune response or as autologous DCs loaded with the tumor-Ag source.

The Phase III pivotal study that allowed FDA approval of ipilimumab to treat unresectable Stage III–IV melanoma patients was in fact designed to determine the safety and efficacy of ipilimumab in combination with BMS-734019 vaccine, a tumor-associated Ag (TAA) gp100-peptide vaccine, versus vaccine or ipilimumab alone (NCT00094653) (7). The main results of that study were that ipilimumab, with or without a vaccine, in comparison to vaccine alone, improved OS of metastatic CM patients. Severe adverse events were observed but most of them were reversible and manageable. In 2015, a pooled analysis was performed of long-term survival data of 1861 patients from Phase II and III studies (NCT00032045, NCT00058279, NCT00077532, NCT00094653, NCT00135408, NCT00261365, NCT00289627, NCT00289640, NCT00324155, and NCT00623766), some of them including combined vaccination with peptides, the majority of patients receiving the 3 mg/kg regimen. Also, data from additional 2,985 patients from an expanded access program were analyzed. A plateau in the survival curve was observed, beginning at approximately 3 years, which was independent of prior therapy or ipilimumab dose, supporting the impact of ipilimumab in long-term survival for advanced CM patients (12).

Recent combination trials of ICKB with peptide vaccines are shown in Table 1. 6MHP, a melanoma vaccine comprised of 6 MHC-II-restricted helper peptides administered subcutaneous (SC) and intradermally (ID) as water-in-oil emulsions with Montanide ISA-51, in combination with pembrolizumab (NCT02515227) or ipilimumab (NCT02385669), is under investigation. NCT03047928 trial will combine nivolumab with a peptide vaccine consisting of PD-L1 and indoleamine 2,3-dioxygenase (IDO) peptides. T-cell reactivity against PD-L1 and IDO in the tumor microenvironment and in the peripheral blood of CM patients with cytotoxic activity has been reported (13). Thus boosting specific T cells that recognize immune regulatory proteins such as IDO and PD-L1 may directly modulate immune regulation. In the protocol, patients will be treated with nivolumab every second week as long as there is a clinical benefit. The PD-L1/IDO peptide vaccine is given from the start of nivolumab and every second week for the first six vaccines and thereafter every fourth week up to 1 year. NCT01176461 tested the side effects of an investigational vaccine consisting of gp100280-288 and NY-ESO-1157-165 MHC-I peptides and adjuvant Montanide ISA-51-VG, combined with escalating doses of antagonist PD-1 mAb BMS-936558. A cohort of patients will not receive the vaccine. Phase I trial results indicated that combination was well tolerated and induced responses lasting up to 140 weeks (14).

Another interesting strategy is being tested in NCT02897765, combining nivolumab with NEO-PV-01, a personalized vaccine therapy designed to target mutated proteins which are present uniquely on an individual’s tumor neoAgs, with poly-ICLC as the adjuvant. Patients will receive 240 mg IV nivolumab every 2 weeks and those patients who have not achieved a complete response to nivolumab alone at week 12 will receive NEO-PV-01^+^ adjuvant SC, in up to four distinct sites while continuing therapy with nivolumab. The study will monitor side effects and Ag-specificity in peripheral CD8^+^ and CD4^+^ T cell responses and tumor biopsies, which will be assessed after treatment.

NCT01302496 studies ipilimumab combined with TriMix vaccine. The TriMix-DC vaccine is a DC-based vaccine that can induce a T-cell repertoire that recognizes the TAA MAGE-A3, MAGE-C2, tyrosinase, and gp100 in an HLA-restricted way, in unresectable stage III-IV melanoma patients (15, 16). To prepare TriMix-Dc vaccine, autologous DCs are coelectroporated with TriMix mRNA (a combination of CD40L, caTLR4, and CD70 encoding mRNA) in combination with one of four TAA mRNAs linked to an HLA-II targeting signal. After electroporation, the four different TriMixDC-MEL cellular constituents (i.e., DCs expressing one of the four Ags) are mixed at equal ratios and cryopreserved until vaccination. The study is complete but results are still unpublished.

NCT02432963 proposes another combination strategy with ICKB and vaccines, testing a modified Vaccinia Virus Ankara Vaccine expressing tumor protein p53 (p53MVA Vaccine) in combination with pembrolizumab, in patients with tumors with overexpression or mutation of p53. Immune monitoring in peripheral blood samples obtained through the protocol will analyze T-cell reactivity to p53, myeloid-derived suppressor cell (MDSC) and Tregs immunosuppressive populations, and other selected subsets including PD-1^+^, PDL-1^+^, and PDL-2^+^ cells.

Finally, in a Phase I/II study (NCT02275416), ipilimumab (3 mg/kg, every 3 weeks for a total of four doses) in combination with GM-CSF (75 µg) and UV1 peptide based-vaccine directed to hTERT is being tested in unresectable Stage III or Stage IV melanoma patients. UV1 vaccine (300 µg) will be administered by injecting ID in the lower abdomen before and between treatments with ipilimumab, thereafter every 4th week up to 28 weeks, and then at weeks 36 and 48. This study will analyze safety and tolerability of the combination and also will measure specific T-cell responses, quality of life, and treatment response by CT scans. A search for potential biomarkers of efficacy and safety studies will be also performed.

#### Cytokines

There are several trials combining ICKB with typical cytokines first assessed in CM patients as non-specific immunotherapy treatments, such as IL-2, IFN-α2b, and GM-CSF (Table 1). Aldesleukin is a recombinant analog of the endogenous cytokine IL-2 that has immunoregulatory and antineoplastic activities. It promotes activation of T, B, and NK cells; however, serious related adverse events were seen upon IL-2 administration (17). IL-2 was approved for treatment of metastatic renal cell carcinoma in 1992 and for metastatic melanoma (MM) in 1998 by FDA. Nowadays, IL-2 monotherapy is not the optimal and standard treatment for both metastatic renal cell carcinoma and MM but efforts to further improve the efficacy of IL-2 therapy are focused on combined therapies. Results from NCT00058279 combining ipilimumab with IL-2 revealed immune-related adverse events. A non-synergistic effect was observed, since the 22% objective response rate observed, results from the additive effect of the expected response rate for each therapy; however, long-term responses were still observed (12). NCT02983045 ongoing study will analyze ICKB in combination with NKTR-214, a prodrug for IL-2, conjugated to six releasable PEG chains. In a preclinical CM mouse model, this molecule showed 20 times preferential activation of CD8^+^ T cells (IL2Rβ) over Treg cells (IL2Rα) in comparison to aldesleukin. In this model, NKTR-214 proved efficacy as a single agent, and long-term immunity when combined with antagonist CTLA-4 mAb, in addition to resistance to tumor rechallenge (18). NCT02748564 Phase Ib/II study will evaluate the safety and tolerability of IL-2 when given in combination with pembrolizumab to patients with advanced CM.

Adjuvant IFN-α2b increases disease-free survival (DFS) although not OS in CM patients but it is accompanied with considerable toxicity (19, 20); it is not universally considered as a gold standard treatment (21). Besides, the optimal dose and duration of IFN-α2b treatment are still unclear (22, 23). IFN-α2b would have several mechanisms of action, from induction of apoptosis in tumor cells to activation of monocytes and macrophages favoring Ag processing. There are several ongoing trials combining ICKB with IFN-α2b or PEG-IFN-α2b (NCT01608594, NCT01708941, NCT02112032, NCT02339324, and NCT02174172). Low doses of cytokine GM-CSF proved to be a strong monocyte attractant and necessary to differentiate monocytes into DCs promoting a T_H_1 response (24); a combination of ipilimumab with this cytokine is also on the way (NCT02009397).

#### Adoptive Cell Therapy

One approach to restore the functionality of effector immune cells is to cultivate autologous tumor infiltrating lymphocyte (TIL) *ex vivo* after tumor resection and infused them back into the patient (25); this is defined as ACT. Combination of ACT with ICKB may counteract any inhibitory immune checkpoint signal from the tumor microenvironment, provided that T cell effectors have been expanded and activated *in vitro* in the presence of tumor Ags previous to treatment (Table 1). NCT02652455 will compare the effect of nivolumab administration prior to tumor resection and *in vitro* culture of TILs. These will be cultivated *ex vivo* with agonist CD137 mAb to augment T cell proliferation and infused them after chemotherapy-induced lymphodepletion of patients. They will be treated *in vivo* with IL-2 to support T cell proliferation. NCT01701674 will study the effect of ipilimumab before leukapheresis, while NCT02027935 will do it afterward.

#### Stimulatory Immune Checkpoints

CD40 is a costimulatory receptor that is essential for activating both innate and adaptive immune systems (26). CD40 binds its ligand CD40L, which is transiently expressed on T cells and other non-immune cells under inflammatory conditions. A wide spectrum of molecular and cellular processes is regulated by CD40 engagement including the initiation and progression of cellular and humoral adaptive immunity. Use of agonist CD40 mAbs with high-affinity fosters activation of APCs (DCs, monocytes, and B cells), leading to stimulation of tumor-specific immune responses. Recently, it was reported in a mouse tumor model that use of agonist CD40 mAb reversed resistance to PD-1, downregulating PD-1 levels in T cells *via* IL-12 production (27). Agonist CD40 mAb APX005M is currently being evaluated in Phase I/II trials in combination with ipilimumab (NCT03123783) or pembrolizumab (NCT02706353) (Table 1). NCT02554812 trial combines avelumab in different cohorts with agonist mAbs toward T cells costimulatory molecules 4-1BB and OX-40 (28) or neutralizing mAb toward M-CSF/CSF1 (macrophage colony-stimulating factor) (29).

#### Toll-Like Receptors (TLRs)/PAMP

Toll-like receptors can detect a broad range of human pathogens, as well as a variety of molecules such as PAMP (pathogen-associated molecular patterns) that indicate tissue damage. This recognition triggers a cascade of innate and adaptive immune responses that fully activate the immune system. Agonist TLR mAbs support this response. It was reported that triggering of TLR3 induces T-cell activation and a strong upregulation of HLA-I and PD-L1 in neuroblastoma and melanoma cells (30, 31). Therefore, ICKB will counteract limitation of the T cell response induced by TLR signaling. Ongoing trials include combinations of PD-1 and CTLA-4 ICKB with agonist TLR3 and TLR9 mAbs (NCT02643303, NCT02644967, and NCT02668770). Trial NCT02981303 will assess Imprime PGG, a β-1,3/1,6 glucan PAMP molecule isolated from the cell wall of a proprietary Saccharomyces, in combination with pembrolizumab (Table 1).

### ICKB Combined with Targeting of Immunosuppressive Molecules/Pathways

#### Other ICKB

Immune checkpoint blockade is also being assessed in combinations with the targeting of other molecules/pathways that promote an immunosuppressive environment (Table 2). For instance, there are trials targeting several ICKB. NCT02743819 trial combines pembrolizumab with ipilimumab in advanced patients which, following treatment with PD-1/PD-L1 mAb, either progress or present stable disease/initial response for more than 24 weeks. NCT02381314 studies in B7-H3 expressing tumors such as CM, the combination of ipilimumab with enoblituzumab, a B7-H3 mAb was designed to improve ADCC by increasing FcR affinity. NCT02460224 analyzes the combination of LAG525 and PDR001, antagonist mAbs for LAG-3 and PD-1, respectively. LAG-3 is an immune checkpoint that binds a non-holomorphic region of the MHC-II molecule and has an important role in the tumor microenvironment. It was reported that soluble-LAG-3 binds to immature DCs, promoting their maturation (32). However, LAG-3 is involved in alternative activation of plasmacytoid DCs in melanoma lesions (33). Interaction of MHC-II on APCs with LAG3 downregulates T-cell proliferation and activation (34). In agreement, LAG-3 mediates resistance to apoptosis on MHC-II expressing melanoma cells (35). LAG-3 is substantially expressed on melanoma TILs, including those with potent immunosuppressive activity. LAG3 was shown to have a synergistic action with the PD-1/PD-L1 axis, critical for releasing an antitumor immune response. In a mouse melanoma model, tumor-specific CD4^+^ effector T-cells showed traits of chronic exhaustion, with high expression levels of PD-1, TIM-3, 2B4, TIGIT, and LAG-3 inhibitory molecules. Blockade with a combination of anti-PD-L1 and anti-LAG-3 mAbs overcame the requirement to deplete tumor-specific Tregs in this model (36). The PD-1 expression on CD8^+^ TILs identified a repertoire of clonally expanded tumor-reactive cells, including mutated neoAg-specific CD8^+^ T-cells; these cells expressed LAG-3 and Tim-3 (37). It has recently been described that ipilimumab expanded T cells in patients with higher expression levels of CD27, intracellular CTLA-4, TIM-3, and LAG-3, which can be taken into account for future combination trials (38).

Adenosine-A2A receptor (A2Ar), an ectonucleotidase that catabolizes the hydrolysis of extracellular adenosine monophosphate (AMP) to adenosine, is a novel metabolic target for ICKB. In preclinical models, it was shown that expression of A2Ar on myeloid cells suppressed T and NK cell responses in the solid tumor microenvironment (39). Also, A2Ar blockade enhanced antitumor activity of PD-1 and CTLA-4 ICKB (40). NCT02655822 is on the way combining PD-1 ICKB with an A2Ar inhibitor molecule.

#### T-Cell Exhaustion

TIM-3 was first identified as a specific T_H_1 receptor. When it binds to galectin-9, it generates an inhibitory signal that results in apoptosis of T_H_1 cells (41). TIM-3 was also described as a marker of T-cell exhaustion (38). TIM-3 is also expressed by NK cells and naive DCs, acting in synergy with TLR signaling to induce inflammation. Expression of TIM-3 in monocytes and macrophages promotes phagocytosis of apoptotic cells through interaction with phosphatidylserine, which enhances Ag cross-presentation (42). Also, TIM-3 binds HMGB1, impairing its recruitment of nucleic acids into endosomes, a key step in the sensing of DNA by the innate immune system, promoting tumor escape (43). Notably, it was shown that PD-1 and Tim-3 limited the expansion of tumor Ag-specific CD8^+^ T cells induced by a melanoma peptide vaccine, as dual blockade enhanced the expansion and cytokine production of vaccine-induced CD8^+^ T cells *in vitro* (44). NCT02817633 and NCT02608268 are ongoing combining ICKB with an antagonist Tim-3 mAb (Table 2).

#### Tumor Immune Microenvironment

NCT02983006 trial is testing the combination of nivolumab with DS-8273a (Table 2). This agonist mAb showed selective targeting of MDSC through TNF-receptor TRAIL-DR2, without affecting other mature myeloid or lymphoid cells (45). As an agonist of TRAIL-DR5 to induce apoptosis in tumor cells, in a Phase I trial DS-8273 as monotherapy was well tolerated but no objective responses were observed, although decreases in MDSC temporally associated with DS-8273a exposure were observed (46).

Indoleamine 2,3-dioxygenase is the first and rate-limiting enzyme involved in tryptophan catabolism, which can halt T-cells growth. In cancer, IDO is expressed within the tumor itself as well as in the tumor microenvironment, where it promotes the establishment of peripheral immune tolerance to tumor Ags. On the tumor side, lymph node CM cells express IDO, recruiting Treg, which is associated with a poor outcome (47). At the tumor microenvironment, IDO promotes MDSC recruitment by a mechanism dependent on Tregs (48); it also inhibits NK cell function along with PGE-2 (49). Activated T cells *in vitro* induce MDSC function through IL-10; these MDSC secrete ARG-1 and IDO and express PD-L1 and MHC-II, leading to upregulation of PD-1 and LAG-3 on T-cells, promoting an immunosuppressive tumor microenvironment (50). In CM patients, high levels of circulating PD-L1^+^ cytotoxic T-cells were associated with increased expression levels of CTLA-4 in Tregs and IDO in MDSC and plasmacytoid DCs. All these parameters were related to a negative outcome, independent of disease stage (51).

It is interesting to note that IDO is an immunogenic protein, therefore, activation of pro-inflammatory IDO-specific CD4^+^ responses may delay or overcome the immunosuppressive actions of IDO, consequence of early expression in maturing APCs; however, IDO-specific Tregs may enhance IDO-mediated immune suppression (47). In mouse melanoma models, IDO is an essential mechanism of resistance to ICKB, including CTLA-4 and PD-1. CTLA-4 blockade combined with IDO inhibitors strongly synergizes to mediate tumor rejection (49). It is postulated that following melanoma infiltration by lymphocytes, upregulation of PD-L1, IDO, and Tregs is regulated by an intrinsic immune mechanism (52). And that combination of CTLA-4, PD-1/PD-L1, and IDO blockade restores IL-2 production and proliferation of CD8^+^ T-cells (53). It was recently reported that melanoma expresses high levels of IDO and galectin-3, which upregulate Tregs, suppressing the expansion of tumor-specific T cells cultivated for ACT, which could be reversed by blockade of IDO and galectin-3 (54). Trials combining ICKB with inhibitors of IDO (NCT02471846, NCT02318277, NCT02327078, and NCT02073123) or galectin-3 (NCT02117362) are currently on the way (Table 2).

NCT02403778 trial proposes the combination of ipilimumab with all-trans retinoic acid (ATRA), a derivative of vitamin A (Table 2). ATRA induces maturation of immunosuppressive MDSCs into myeloid cells (55, 56); this was shown to be of benefit in a lung cancer vaccine and ACT for sarcomas (57, 58). Thus, this combination is designed to decrease MDSCs and differentiate immature monocytes into mature DCs and increase tumor Ag-specific T-cell responses. In this trial, ATRA single-arm versus ATRA with ipilimumab combined-arm will be compared.

Finally, other combinations of ICKB and targeting the tumor microenvironment include antagonist mAbs for M-CSF/CSF1 (NCT02807844) or its receptor MCSFR/CSF1R (NCT02452424 and NCT02880371) (Table 2). Interaction of α4β1 integrin from extracellular matrix with MCSF receptor leads to the activation of Rac2 and regulation of macrophage toward a M2 immunosuppressive phenotype (59). In a melanoma mouse model, targeting of CSF1R on MDSCs overcomes resistance to IDO-expressing melanoma cells (60).

### Other Combinations with Immunomodulatory mAbs

There are several oncolytic viruses that have shown to promote an immunogenic cell death leading to a T_H_1 response; combination with ICKB is aimed to sustain in time this tumor microenvironment (61). Talimogene laherparepvec (T-VEC, Imlygic) is a genetically modified, attenuated, herpes simplex virus type 1 designed to promote an antitumor response through selective viral replication in tumor cells and stimulation of systemic antitumor immunity through GM-CSF (62). This was the first oncolytic viral therapy to be approved by the FDA in 2015 for intralesional treatment of unresectable lesions in patients with melanoma recurrent after the initial surgery. The combination of T-VEC with ipilimumab in a Phase I trial proved to be safe and appeared to have greater efficacy than single agents (NCT01740297) (63). NCT02263508 is a Phase 1b/3 study that will assess the combination of talimogene laherparepvec with pembrolizumab in unresectable CM patients. Combination studies with other oncolytic virus include NCT02272855 trial, which will analyze CTLA-4 ICKB with HF10, an oncolytic virus that has shown to induce angiogenesis and affluence of CD8^+^ T-cells at the tumor microenvironment (64). Finally, in the NCT03003676 study the combination of pembrolizumab with ONCOS-102, an engineered oncolytic Adenovirus expressing GM-CSF, will be analyzed in CM patients that have progressed to the PD-1 blockade.

The NCT01986426 study is designed to assess the safety, tolerability, and efficacy of different intratumoral dosing regimens of LTX-315; a lytic-peptide that induces immunogenic cell death; it will be assessed as monotherapy or in combination with ipilimumab or pembrolizumab. It was shown in mouse models that this peptide overcomes tumor resistance to CTLA-4 ICKB (65) (Table 1).

The NCT02302339 study will examine the effectiveness and safety of glembatumumab vedotin as monotherapy and in combination with either nivolumab or pembrolizumab (Table 3). Glembatumumab vedotin mAb is conjugated to the cytotoxic drug monomethyl auristatin E. This mAb targets glycoprotein NMB, expressed on the surface of tumor cells, releasing the drug and inducing tumor cell death. Combinations with immunotherapy include pembrolizumab/nivolumab and varlilumab, an agonist mAb of the T-cell costimulatory receptor CD27. NCT02076633 trial also examines conjugated mAbs for treatment of metastatic CM patients; L19IL2 targets melanoma cells through HDAC4 and it is conjugated to IL-2; instead L19TNF is conjugated to TNFα, exerting its major effects *via* a preferential toxicity for the endothelial cells of the tumor-associated vasculature, therefore, increasing an antitumor immune response. Preclinical data suggest that intratumoral administration of these conjugates can be more effective.

Another approach explores the synergy of two agonist mAbs targeting the T-cells costimulatory molecules OX40 and 41BB (25, 66) (NCT02315066) (Table 3). Finally, NCT02714374 ongoing trial is combining GL-ONC1, a genetically modified oncolytic vaccinia virus, with eculizumab, a neutralizing C5 mAb, with the goal that GL-ONC1 remains in the body long before being cleared by the immune system.

## Discussion

Combined immunotherapy involving mAbs and other immunomodulatory strategies is an emerging field. There is no still any such combination therapy approved. The immune system should be considered as one interrelated signaling network where targeting different points may act synergistically to promote anticancer effects. Proper immune stimulation and blockade of immunosuppression can be seen as a “push and release” strategy, where both are critical for the efficacy of an anti-cancer immunotherapy (Figure 1). ICKB is currently being assessed in combination with immune stimulatory strategies, such as vaccination, cytokines, ACT, stimulatory immune checkpoint agonists, and targeting of TLRs. Other approaches combine ICKB with targeting of several immune suppressive mechanisms, such as blocking other immune checkpoints, T-cell exhaustion inhibition, and promotion of an antitumor microenvironment.

The idea of combining ICKB with immunomodulatory strategies such as vaccines is very attractive, given that in some cases amplification of Ag-specific T cells, as well as the induction of antibodies recognizing tumor Ags are observed after vaccination. Blocking of immune checkpoints may result in effector T cells that could attain potent tumor destruction more potently; a useful T helper function and modulation of Tregs will result in the expansion of cytotoxic T cell effectors. However, vaccines, in general, have shown a low 10–15% rate of clinical responses, with still a lack of efficacy to eradicate tumor masses and avoid further dissemination in metastatic patients (67). Most of the clinical trials revised in this work are being assessed in advanced CM patients, thus immune suppression, both systemic and local, can be hard to overcome even with ICKB, since there are patients that do not respond at all. Vaccination strategies may be more useful when administered in the adjuvant setting to control tumor relapse in high-risk stage II-III CM patients (68), and thus, their combination with ICKB may hold the promise of durable clinical benefit avoiding metastases to distant organs and achieving prolonged OS.

Assessment of clinical responses in ICKB cancer treatments can be challenging since traditional Response Evaluation Criteria In Solid Tumors, RECIST, may underestimate the actual response that can be delayed and atypical, as evidenced in patients treated with ICKB (69). The immune-related response criteria have been established to allow patients to continue treatment after the first progression until a new progression is presented, given the chance of eventual clinical benefit to more patients (70). It should be taken into account that there are both constitutive and acquired IKCB resistance mechanisms compromising treatment outcome (71–73).

Identification of early predictors of response is desirable to identify patients that would benefit the most and avoid unnecessarily prolonged treatments. Regarding ICKB biomarkers at the local level, an association of PD-L1 expression in pretreatment tumor biopsies with objective response to anti-PD-1/PD-L1 therapy has been observed given the constant finding that PD-L1 expression is enriched in anti-PD-1/PD-L1 therapy responders in several tumors (74). Weighted-average ORR across several studies for patients whose tumors were tested for PD-L1 is 29%; if the tumor expresses PD-L1, these increases to 48%. However, a significant proportion of responding patients with PD-L1 negative-tumors were observed. Also, high-density infiltration of CD8 T-cells in tumor biopsies has been associated with PD-L1 expression in tumor cells; it was associated independently with an improved prognosis, with increased time to development of brain metastases in CM patients (75). Also, it was recently reported that PD-L2 expression in metastatic CM was associated with immune infiltration and a better prognosis independently of therapy of choice (76). Other proposed biomarkers for selecting which patients are likely to benefit from cancer immunotherapies are the tumor mutational load and microsatellite in the stability. CM is a tumor with a high rate of mutation (1), and thus with a higher probability of neoAg generation, increasing the number of immunogenic structures that could stimulate a more potent and broad repertoire of antitumor immune effectors. In the same way, microsatellite instability is a frequent event in CM which accounts for tumor immunogenicity, contributing to making of CM a pathology suitable for immunotherapy (77).

At the peripheral level, serum IL-8 concentrations actually reflect tumor burden (78). It was recently reported that measurement of serum IL-8 levels 2–3 weeks following starting therapy can predict response and OS in metastatic CM patients treated with PD-1 ICKB, even before imaging evaluation (79). Also, there are recent publications by two independent groups which reported that assessment of circulating cell-free DNA from tumors in CM patients receiving ICKB treatment is an accurate predictor of tumor response, PFS and OS, as patients with elevated ctDNA on therapy had a poor prognosis (80, 81).

In the combination of several immunotherapy strategies, identifying which patients are likely to respond to therapy will be even more challenging. This is given the complexity of the immune system, and the limited understanding of its regulation and multiple interactions between immune cells, immune-modulating molecules, tumor cells, and other compartments of the tumor microenvironment, such as the lymphatic and blood systems.

## Conclusion

Monoclonal antibodies have gained evidence of their effectiveness for cancer treatment. This seems to be the tip of an iceberg, as we are learning that not only targeting the tumor but also modulating the immune response, may be a powerful way to achieve long-term clinical responses. In this way, mAbs can be again considered “magic bullets” targeting molecules with different immunomodulatory effects. We have revisited most of the current clinical trials that explore combined use of immunomodulatory mAbs with different immunotherapeutic approaches, with the aim to improve and/or potentiate clinical responses in CM patients. This is an exciting and expanding research field that is rapidly spreading to other tumor types.

## Author Contributions

MA and MB: conception and design of the review; manuscript writing. JM: conception and design of the review.

## Conflict of Interest Statement

The authors declare that the research was conducted in the absence of any commercial or financial relationships that could be construed as a potential conflict of interest.

## Abbreviations

Ags, antigens; APCs, Ag-presenting cells; CM, cutaneous melanoma; CTLA-4, cytotoxic T-lymphocyte antigen 4; DCs, dendritic cells; DFS, disease-free survival; ICKB, immune checkpoint blockade; ID, intradermally; IV, intravenous; IDO, indoleamine 2,3-dioxygenase; MDSC, myeloid-derived suppressor cell; MM, metastatic melanoma; mAb, monoclonal antibodies; neoAgs, neoantigens; OS, overall survival; PAMP, pathogen-associated molecular patterns; PD-1, programmed cell death-1/CD279; SC, subcutaneous; Tregs, regulatory T cells; TLR, toll-like receptors; TAA, tumor-associated Ag; TIL, tumor infiltrating lymphocytes.
